# Could semiquantitative analysis of real-time ultrasound elastography distinguish more liver parenchyma alterations of nonalcoholic fatty liver disease in patients with polycystic ovary syndrome?

**DOI:** 10.20945/2359-3997000000119

**Published:** 2019-03-18

**Authors:** Na Di, Xinchuan Zhou, Yaxiao Chen, Xiaomiao Zhao, Lin Li, Linlin Jiang, Baoming Luo, Xiaoli Chen, Dongzi Yang

**Affiliations:** 1 Sun Yat-sen University Sun Yat-sen University Sun Yat-sen Memorial Hospital Department of Ultrasound Guangzhou China Department of Ultrasound, Sun Yat-sen Memorial Hospital of Sun Yat-sen University, Guangzhou, China; 2 Sun Yat-sen University Sun Yat-sen University Sun Yat-sen Memorial Hospital Department of Obstetrics and Gynecology Guangzhou China Department of Obstetrics and Gynecology, Sun Yat-sen Memorial Hospital of Sun Yat-sen University, Guangzhou, China

**Keywords:** Polycystic ovary syndrome, nonalcoholic fatty liver disease, real-time ultrasound elastography, semiquantitative analysis, metabolic disturbances

## Abstract

**Objective:**

Nonalcoholic fatty liver disease is the commonest diffuse liver disease, of which women with polycystic ovary syndrome are at an increased risk. The aim of the present study was to assess the diagnostic value of the semiquantitative strain parameters of real-time ultrasound elastography for nonalcoholic fatty liver disease in patients with polycystic ovary syndrome.

**Subjects and methods:**

Thirty-five polycystic ovary syndrome patients with nonalcoholic fatty liver disease, 70 polycystic ovary syndrome patients without nonalcoholic fatty liver disease, and 70 healthy female controls of reproductive age were included. All participants underwent ultrasonic examination and semiquantitative analysis of real-time ultrasound elastography of the liver.

**Results:**

Main semi quantitative strain parameters, such as average strain value, differed significantly among groups polycystic ovary syndrome with nonalcoholic fatty liver disease, polycystic ovary syndrome without nonalcoholic fatty liver disease, and control (87.02 ± 10.16 vs. 96.31 ± 11.44 vs. 104.49 ± 7.28, p < 0.001). Clinical and laboratory parameters differed significantly between the two subgroups with low or high average strain value. For diagnostic value of average strain value for elevated aminotransferase, the area under the curve was 0.808 (range 0.721-0.895). In multiple linear regression analysis, polycystic ovary syndrome, waist circumference, and metabolic syndrome were stand-alone independent factors associated with average strain value among subjects without nonalcoholic fatty liver disease.

**Conclusion:**

Semiquantitative real-time ultrasound elastography analysis could distinguish liver parenchyma alterations in patients with polycystic ovary syndrome more sensitively. The diagnostic value of the proposed method for nonalcoholic fatty liver disease need further research.

## INTRODUCTION

Nonalcoholic fatty liver disease (NAFLD) is the commonest diffuse liver disease, comprising the spectrum of liver damage from simple hepatic steatosis to nonalcoholic steatohepatitis to advanced fibrosis and cirrhosis, in patients without significant alcohol consumption and any other cause of liver diseases. The worldwide prevalence of NAFLD, which is 5%-58% in the general population, has increased over time and has become a major public health problem (1). In the Chinese population from Hong Kong, the prevalence of NAFLD is as high as 42% according to a 2015 report (2). NAFLD is also used as a marker of severe metabolic disorders.

Polycystic ovary syndrome (PCOS) is the most common endocrine disorder among women of reproductive age, and the prevalence varies from 5% to 20% (1,3). It is also a metabolic disease according to high prevalence of the accompanying metabolic disturbances, which may pose a high long-term risk of cardiovascular diseases for patients with PCOS (4). Women with PCOS are at an increased risk of NAFLD as reported previously: the prevalence of NAFLD varies from 23.8% to 86.79% among women with PCOS (5,6). Accordingly, it has been suggested that women with PCOS should be screened for liver disease more actively (7).

Although a liver biopsy is still considered the gold standard for detection of a liver disease such as fibrosis, this procedure is invasive and is associated with possible problems such as bleeding and severe pain, sampling errors, and interobserver variability (8). Computed tomography (CT) or magnetic resonance imaging (MRI) can quantify liver fat, but CT involves ionizing radiation, and both methods are costly (9). Conventional ultrasonography has been used for detection of NAFLD in daily practice, but its accuracy is limited by subcutaneous fat thickness of patients and the operator's experience. Furthermore, it cannot provide information about mechanical properties of the tissue. It is impractical to use these methods for assessment and frequent monitoring of NAFLD in PCOS patients of reproductive age. Real-time tissue elastography (RTE), one of the ultrasound elastography technologies, has been rapidly evolving in the last decade, being used for diagnosis of focal lesions in various organs. According to recent reports, RTE is also useful for assessment of fibrosis in patients with chronic liver diseases (10,11). With the improvements in RTE, semiquantitative analysis of strain was developed. Parameters including the average strain value (MEAN) of RTE are reported to be useful for early diagnosis and staging of liver fibrosis (12–18).

On the other hand, application of RTE with semiquantitative parameters to NAFLD is rarely reported, never in PCOS women of reproductive age. The aims of our study were to analyze semiquantitative strain parameters of RTE of the liver in PCOS patients with or without NAFLD as compared to healthy controls and then to evaluate the diagnostic value of these parameters for NAFLD in patients with PCOS.

## SUBJECTS AND METHODS

### Sample size estimate

No previous study had reported the efficacy of RTE in diagnose of NAFLD in patients with PCOS. According to report about RTE for assessment of liver fibrosis in chronic hepatitis B (18), a sample size of 5 in each group has a power of 0.949 at 5% significance to detect a difference of 6.39 in the value of MEAN between cases and controls calculated by PASS 11(NCSS LLC, USA). We recruited more than 5 participants for each group. A total of 35 PCOS patients with NAFLD, 70 PCOS patients without NAFLD, and 70 healthy female controls aged 20 to 40 years were recruited in our Hospital from May 27, 2015, to November 31, 2015.

### Patients and controls

The study was approved by the Institutional Review Board of our Hospital. All participants provided written informed consent. The Chinese Clinical Trial Registry (http://www.chictr.org/en/) number is ChiCTR-OOC-15006452. Detailed data on all participates were deposited in the Research Electronic Data Capture (REDCap) databases.

PCOS was diagnosed according to the Rotterdam 2003 criteria (19), two of the following three criteria had to be met: 1) oligomenorrhea and/or anovulation, 2) clinical and/or biochemical hyperandrogenism, 3) polycystic ovaries (PCO) (presence in each ovary of 12 or more follicles measuring 2 to 9 mm in diameter and/or increased ovarian volume: more than 10 mL). NAFLD was diagnosed according to conventional criteria (conventional ultrasonography) (20). Controls were defined as healthy non pregnant, amenorrheic women without endocrine disorders, PCO, and NAFLD.

Women were excluded from the study if they had a medical history that included other known liver diseases, ovarian surgery, a malignant tumor, tobacco smoking, and alcohol drinking, other endocrine diseases, or a severe cardiocerebrovascular disease or if they were taking medication that may affect metabolism, blood pressure, or liver function during the last 3 months.

### Bioclinical tests

The medical history including menstrual regularity, medications, and past diseases was recorded. All anthropometric data were measured and recorded including waist circumference (WC), hip circumference (HC), waistline/hipline ratio (WHR), body mass index (BMI), acne score, modified Ferriman-Gallwey score (mFG) for body hair evaluation, systolic blood pressure (SBP), and diastolic blood pressure (DBP).

Basal follicle-stimulating hormone (FSH), luteinizing hormone (LH), prolactin (PRL), estradiol (E2), and total testosterone (TT) were quantified by chemiluminesce Immunoassay on an automatic biochemistry analyzer (DXI800, Beckman COULTER, Inc. USA) during the early follicular phase or first 3 days of progestin withdrawal bleeding. Anti-Müllerian hormone (AMH) and sex hormone-binding globulin (SHBG) were analyzed by an enzyme-linked immunosorbent assay (ELISA) on a ELx808 plate reader (Biotek Instruments, Inc. USA). Serum transaminases, plasma glucose, insulin, and lipid parameters were measured using standard methods on an automatic chemiluminescence immunoassay system (ADVIA Centaur xp, SIEMENS, Germen).

Oligomenorrhea/amenorrhea and PCO were defined according to the revised Rotterdam criteria. Biochemical hyperandrogenism (bHA) was defined as a total testosterone level of 2.39 nM or greater (21). Hirsutism was defined as mFG ≥ 5 as previously reported (22). The acne score was measured according to a previous report (23). Homeostatic model assessment for insulin resistance (HOMA-IR) was calculated as fasting blood glucose (FBG) ×fasting insulin (FIN) ÷ 22.5. Insulin resistance (IR) was defined as HOMA-IR ≥ 2.14 and FIN ≥ 12.6 mIU/L (24,25). Impaired fasting glucose tolerance (FGT), impaired glucose tolerance (IGT) and type 2 diabetes mellitus (DM) were diagnosed according to 2006 WHO criteria (26). Hyperlipidemia was defined as cholesterol (CHOL) ≥ 6 mM, triglycerides (TG) ≥ 2.3 mM, or low-density lipoprotein cholesterol (LDL-C) ≥ 3.6 mM. Elevated aminotransferase was defined as alanine transaminase (ALT) or aspartate transaminase (AST) ≥ 40 U/L. Central obesity was defined as the waistline ≥ 80 cm. Elevated blood pressure (EBP) was defined as SBP ≥ 130 mmHg or DBP ≥ 85 mmHg. Metabolic syndrome (MS) was diagnosed according to NCEP-ATP III criteria (27).

### Ultrasound examination and RTE

Transvaginal ultrasonography was performed in the early follicular phase if menses were regular or randomly if menses were irregular. Antral follicle count (AFC) was recorded.

All procedures were performed by a single operator with 10 years of experience in transabdominal ultrasonography who had specialized in elastography for the last 5 years. The operator was blinded to the clinical data of all the participants.

The ultrasonic examination (US) was performed by means of HI VISION PREIRUS (Hitachi Medical, Tokyo, Japan), which has both conventional ultrasonography and RTE capabilities. Conventional US was performed with a 3.5 MHz probe. A7-3MHz linear transducer (L52) was used for RTE.

RTE was performed as reported previously (16). The region of interest window was set to 1 cm under the Glisson's capsule in the right lobe, avoiding bile ducts and bile cysts. The pressure causing liver tissue strain came from subjects’ own heartbeat. The RTE software (EZU-TESH1, Hitachi) was used to generate histograms. Three effective acquisitions were performed for each patient. The mean of the total of three measurements served as a representative strain value. We measured 11 characteristic parameters of elastography imaging including MEAN in the range of 0-255 arbitrary units (a.u.) and the area ratio of a low-strain region (AREA, %) as the main characteristics of liver stiffness. Standard deviation of the relative strain value (SD) was also determined.

### Data analysis

The results are presented as mean ± standard deviation or as a rate. The one-sample Kolmogorov-Smirnov test was used to determine whether the distribution of variants was normal. MEANs were compared among the groups using the *t* test or one-way analysis of variance (ANOVA). When equal variances were not assumed, variants were compared by nonparametric tests (Mann-Whitney *U* test or Kruskal-Wallis test). Categorical data were compared among the groups using the chi-squared test. Receiver operating characteristic (ROC) curves were generated to analyze the diagnostic value of MEAN for elevated aminotransferase. Multiple linear regression analysis was used to access the correlation between MEAN or AREA and clinical parameters. These analyses were performed in the SPSS software, version 13.0 for Windows (SPSS, Inc., Chicago, IL, USA), and statistical significance was assumed at *p* < 0.05.

## RESULTS

BMI, WC, WHR, SBP, DBP, FBG, two-hour glucose (2hGLU), FIN, HOMA-IR, TG, ALT, and AST levels significantly decreased in the following order: group PCOS with NAFLD > group PCOS without NAFLD > control group (*p* < 0.05). HDL-C levels showed an inverse trend among the three groups (*p* < 0.05). LDL-C, apoB, AMH, LH, TT, mFG, menstrual cycles, and AFC were much higher in the PCOS groups than in the control group (*p* < 0.05), without significant difference between the two PCOS groups. Group PCOS without NAFLD had a significantly higher level of apoA than did group PCOS with NAFLD and control group (*p* < 0.05) (Table 1).

**Table 1:** Baseline clinical characteristics and laboratory parameters of all subjects

	PCOS With NAFLD	PCOS without NAFLD	Control	P_1_	P_2_	P_3_
**N**	**35**	**70**	**70**			
Age^a^	29 ± 4.41	28.83 ± 4.51	28.97 ± 4.71	NS	NS	NS
BMI^a^ (kg/m^2^)	25.41 ± 3.40	22.81 ± 2.67	21.15 ± 2.61	< 0.001	< 0.001	< 0.001
WC^a^ (cm)	86.23 ± 7.94	77.80 ± 8.21	72.91 ± 8.53	< 0.001	< 0.001	< 0.001
WHR^a^	0.8805 ± 0.0528	0.8337 ± 0.0619	0.8013 ± 0.0735	0.001	< 0.001	0.004
SBP^a^ (mmHg)	122.68 ± 12.79	117.18 ± 10.38	111.01 ± 9.48	0.021	< 0.001	< 0.001
DBP^a^ (mmHg)	79.89 ± 9.51	75.85 ± 8.96	69.66 ± 7.14	0.033	< 0.001	< 0.001
FBG^b^ (mmol/L)	5.28 ± 0.83	4.91 ± 0.45	4.72 ± 0.31	0.007	< 0.001	0.012
2hGLU^b^	7.75 ± 2.39	6.50 ± 1.94	5.53 ± 0.93	0.005	< 0.001	< 0.001
FIN^b^ (mIU/L)	25.39 ± 13.95	14.15 ± 6.68	9.20 ± 5.51	< 0.001	< 0.001	< 0.001
HOMA-IR^b^	6.01 ± 4.68	3.07 ± 1.64	1.96 ± 1.34	< 0.001	< 0.001	< 0.001
CHOL^b^ (mmol/L)	5.01 ± 1.35	4.74 ± 0.74	4.59 ± 0.72	NS	NS	NS
TG^b^ (mmol/L)	1.92 ± 1.02	1.25 ± 0.72	0.81 ± 0.28	< 0.001	< 0.001	< 0.001
HDLC^b^ (mmol/L)	1.21 ± 0.25	1.53 ± 0.37	1.44 ± 0.24	< 0.001	< 0.001	NS
LDLC^b^ (mmol/L)	3.25 ± 1.08	2.90 ± 0.65	2.55 ± 0.67	NS	< 0.001	0.002
apoA^b^ (g/L)	1.36 ± 0.33	1.57 ± 0.36	1.40 ± 0.23	0.002	0.002	0.010
apoB^b^ (g/L)	0.88 ± 0.24	0.79 ± 0.21	0.70 ± 0.12	NS	0.001	0.017
ALT^b^ (U/L)	27.44 ± 16.25	15.52 ± 4.50	11.03 ± 4.89	0.001	< 0.001	< 0.001
AST^b^ (U/L)	39 ± 30.91	14.69 ± 8.74	15.17 ± 3.19	< 0.001	< 0.001	0.030
AMH^b^ (ng/dL)	9.99 ± 4.67	9.67 ± 4.52	4.52 ± 3.16	NS	< 0.001	< 0.001
TT^a^ (nmol/L)	1.9355 ± 0.7381	1.80 ± 1.1471	1.2114 ± 0.5210	NS	< 0.001	< 0.001
PRL^a^ (ng/mL)	15.11 ± 7.67	14.53 ± 7.80	15.09 ± 6.81	NS	NS	NS
FSH^a^ (mIU//mL)	6.89 ± 1.87	6.50 ± 2.11	7.84 ± 2.55	NS	NS	0.001
LH^b^ (mIU//mL)	7.53 ± 4.61	9.12 ± 7.30	4.23 ± 1.53	NS	< 0.001	< 0.001
mFG^b^	3.10 ± 3.29	3.78 ± 3.93	0.01 ± 0.12	NS	< 0.001	< 0.001
Acne score^b^	0.1667 ± 0.3791	0.4706 ± 0.7025	0.4265 ± 0.6063	0.039	NS	NS
Menstrual cycle Shortest^b^ (day)	51.52 ± 35.63	42.87 ± 23.89	27.70 ± 2.51	NS	< 0.001	< 0.001
Menstrual cycle Longtest^b^ (day)	108.07 ± 79.06	72.62 ± 45.56	31.68 ± 2.84	NS	< 0.001	< 0.001
AFC^b^	35.32 ± 12.47	31.15 ± 9.45	14.49 ± 3.28	NS	< 0.001	< 0.001

Values are mean ± standard.

P_1_: Comparison between PCOS with and without NAFLD Groups. P_2_: Comparison between PCOS with NAFLD and control Groups. P_3_: Comparison between PCOS without NAFLD and control Groups.

a Compared by T test or one way ANOVA;

b Compared by Nonparametric Test (Mann Whitney U test or Kruskal-Wallis test).

**Figure 1:**
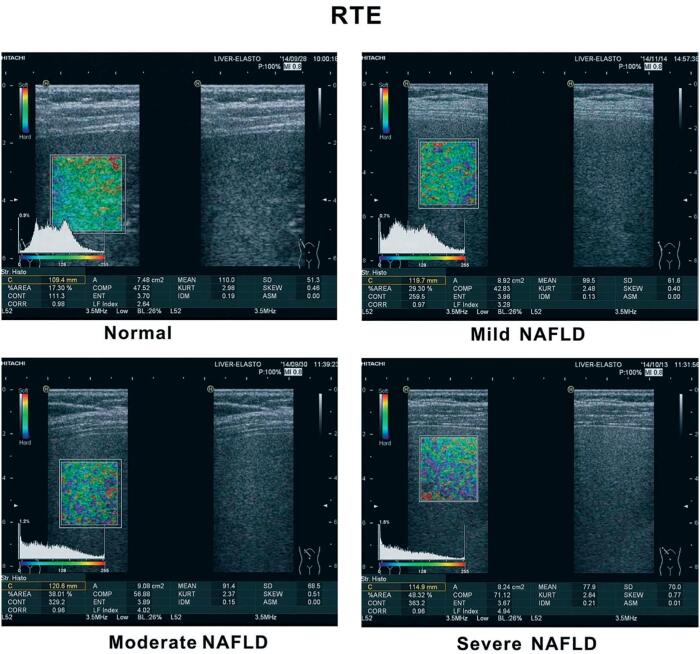
RTE for livers.

**Figure 2:**
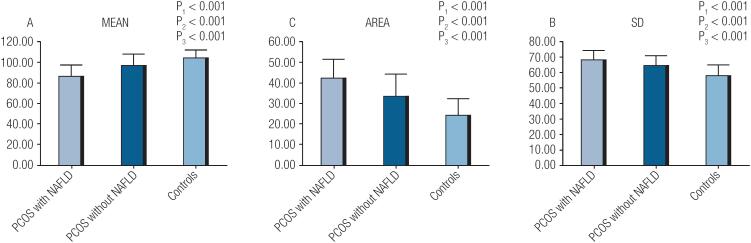
Comparison of RTE parameters between groups PCOS with NAFLD, PCOS without NAFLD, and control. Column bar graphs show significant differences of MEAN, SD, AREA between groups. There were significant trend from PCOS with NAFLD group, PCOS without NAFLD group to control group. P1: Comparison between PCOS with and without NAFLD Groups. P2: Comparison between PCOS with NAFLD and control Groups. P3: Comparison between PCOS Without NAFLD and control Groups.

RTE for livers are shown in Figure 1. The MEAN value was the lowest in group PCOS with NAFLD, intermediate in group PCOS without NAFLD, and highest in the control group (87.02 ± 10.16 vs. 96.31 ± 11.44 vs. 104.49 ± 7.28, *p* < 0.001).The AREA value was the highest in group PCOS with NAFLD, intermediate in group PCOS without NAFLD, and lowest in the control group (42.49 ± 9.41 vs. 33.58 ± 10.91 vs. 24.55 ± 8.14, *p* < 0.001).SD values were significantly different among the three groups in the same descending order as for AREA (68.78 ± 5.21 vs. 64.08 ± 6.73 vs. 58.25 ± 6.58, *p* < 0.001) (Figure 2).

To validate the clinical utility of MEAN, all subjects were subdivided into two subgroups according to MEAN levels. The cutoff value of MEAN (< 91.7) was set to the 5th percentile of the average relative strain value in 70 healthy controls. All 175 subjects were subdivided into group A (MEAN value < 91.7,49 cases) and group B (MEAN value ≥ 91.7, 126 cases).

BMI, WC, WHR, SBP, DBP, FIN, FBG, HOMA-IR, TG, LDL-C, apoB, ALT, AST, AMH, mFG, menstrual cycles, and AFC were significantly higher in group A than in group B (*p* < 0.05). There were no differences in age, 2hGLU, CHOL, apoA, acne score, and other endocrine parameters between the two groups (Table 2).

**Table 2:** Comparison of clinical characteristics between the groups formed on the basis of the cutoff value of MEAN

	Group A (MEAN < 91.7)	Group B (MEAN ≥ 91.7)	*p*
**N**	**49**	**126**	
Age^a^	29.31 ± 4.47	28.77 ± 4.59	NS
BMI^a^	24.84 ± 2.73	21.80 ± 2.93	< 0.001
WC^a^	84.73 ± 8.70	74.65 ± 8.35	< 0.001
WHR^a^	0.8683 ± 0.0644	0.8147 ± 0.0682	< 0.001
SBP^a^	120.90 ± 10.20	113.68 ± 11.05	< 0.001
DBP^a^	78.73 ± 7.53	72.28 ± 9.12	< 0.001
FBG^b^	5.20 ± 0.76	4.801 ± 0.39	< 0.001
2hGLU^b^	7.51 ± 2.20	7.31 ± 2.49	NS
F IN^b^	22.66 ± 13.12	11.26 ± 6.32	< 0.001
HOMA-IR^b^	5.36 ± 4.27	2.43 ± 1.53	< 0.001
CHOL^a^	4.83 ± 1.06	4.70 ± 0.82	NS
TG^b^	1.72 ± 0.99	1.01 ± 0.58	< 0.001
HDLC^a^	1.30 ± 0.35	1.47 ± 0.30	0.002
LDLC^a^	3.07 ± 0.92	2.73 ± 0.73	0.017
apoA^a^	1.43 ± 0.36	1.457 ± 0.30	NS
apoB^a^	0.84 ± 0.21	0.75 ± 0.19	0.013
ALT^b^	22.904 ± 14.23	13.04 ± 5.61	< 0.001
AST^b^	28.07 ± 27.04	15.82 ± 8.79	0.025
AMH^a^	9.79 ± 4.82	6.85 ± 4.53	0.001
TT^a^	1.74 ± 0.62	1.53 ± 1.00	NS
PRL^a^	13.75 ± 7.96	15.23 ± 7.11	NS
FSH^a^	6.87 ± 2.14	7.22 ± 2.40	NS
LH^a^	7.89 ± 7.42	6.43 ± 4.78	NS
mFG^a^	3.41 ± 3.53	1.64 ± 3.18	0.002
Acne score^a^	0.4091 ± 0.7256	0.3934 ± 0.6106	NS
Menstrual cycle Shortest^b^ (day)	48.93 ± 26.01	35.69 ± 22.04	< 0.001
Menstrual cycle Longtest^b^ (day)	96.86 ± 69.07	50.12 ± 37.50	< 0.001
AFC^a^	31.9 ± 12.68	21.86 ± 10.73	< 0.001

Values are mean ± standard.

a Compared by T test or one way ANOVA.

b Compared by Nonparametric Test (Mann Whitney U test or Kruskal-Wallis test).

Prevalence of endocrine disorders and metabolic disturbances was obviously higher in group A than in group B. The prevalence of oligomenorrhea, PCO, PCOS, IR, IFG, hyperlipidemia, central obesity, EBP, and MS was significantly higher in group A than in group B (*p* < 0.05). The prevalence of elevated aminotransferase was also higher in group A (*p* < 0.05). The prevalence of IGT and DM was slightly higher in group A (*p* > 0.05). Nevertheless, there were no differences in the prevalence of HA and hirsutism between the two groups (Table 3).

**Table 3:** Comparison of frequency of endocrine disorders and metabolic disturbances between the groups formed on the basis of the cutoff value of MEAN

	Group A (MEAN < 91.7)	Group B (MEAN ≥ 91.7)	X2	P
**N**	**49**	**126**		
Oligomenorrhea	44 (89.80)	53 (42.06)	32.536	< 0.001
PCO	46 (93.88)	57 (45.24)	32.960	< 0.001
bHA	17 (34.69)	40 (31.75)	0.140	NS
Hirsutism	11 (22.45)	33 (26.19)	0.262	NS
PCOS	46 (93.88)	55 (43.65)	36.469	< 0.001
IR	41 (83.67)	39 (30.95)	39.515	< 0.001
IFG	3 (6.12)	1 (0.79)	4.485	< 0.05
IGT	11 (22.45%)	26 (20.63)	0.070	NS
DM	2 (4.08)	4 (3.17)	0.088	NS
Hyperlipidemia	19 (38.77)	22 (17.46)	8.935	< 0.05
Central obesity	39 (79.59)	33 (26.19)	41.547	< 0.001
EBP	14 (28.57)	17 (13.49)	5.504	< 0.05
MS	17 (34.69)	6 (4.76)	27.689	< 0.001
Elevated aminotransferase	9 (18.37)	2 (1.59)	16.864	< 0.001

Values are number (%).

**Figure 3:**
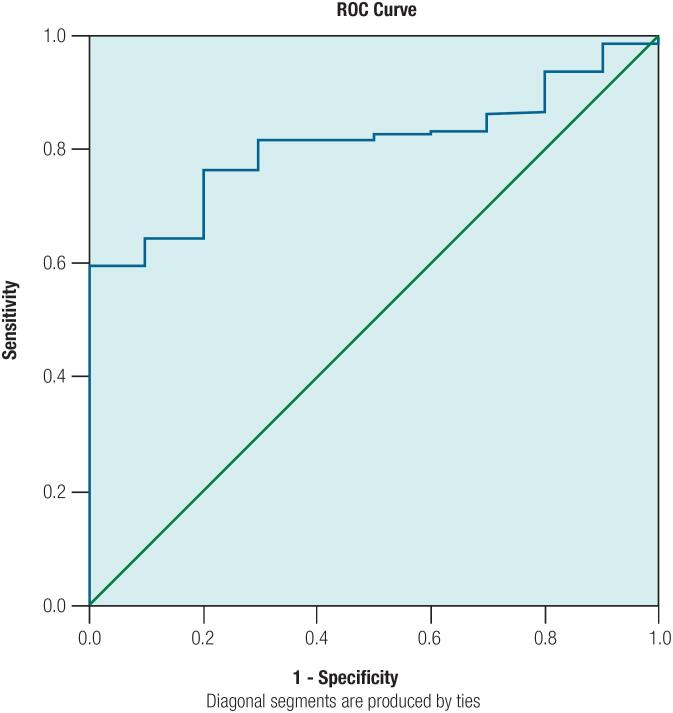
ROC curves for the diagnostic value of MEAN for elevated aminotransferase. ROC curves for MEAN value exhibit reasonable overall performance to discriminate patients with and without elevated aminotransferase.

Receiver operating characteristic (ROC) curves were generated to analyze the diagnostic value of MEAN for elevated aminotransferase: the area under the curve was 0.808 (range 0.721-0.895). With the MEAN level of 91.7 as the cutoff value, the sensitivity and specificity were 0.762 and 0.8 respectively, with the Youden index of 0.562 (Figure 3).

In multiple linear regression analyses, the MEAN value was a dependent variable. Independent variables included AGE, BMI, WC, WHR, mFG, FIN, FBP, HOMA-IR, AMH, CHOL, TG, LDL-C, HDL-C, apoA, apoB, ALT, AST, SBP, DBP, MS, and PCOS. The results showed that only PCOS, WC, and MS were stand-alone independent factors associated with the MEAN value among the 140 subjects without NAFLD (*p* < 0.05).

When AREA was a dependent variable (instead of MEAN), PCOS, WC, and MS were also in the list of stand-alone independent factors (*p* < 0.05).

## DISCUSSION

NAFLD is related to obesity, MS, IR, DM, and other metabolic disorders. These metabolic disorders are more frequent among patients with PCOS than among normal individuals. Studies confirmed that patients with PCOS are at a higher risk of NAFLD than controls are (5,6), and this finding is receiving more attention from gynecologists.

RTE is an excellent supplement to conventional ultrasonography. Nonetheless, it is mainly used for diagnosis of focal lesions with impressive accuracy in daily practice. In RTE, elastic strain is a relative indicator of texture stiffness. Compared to other elastographic methods such as transient elastography (TE), the use of RTE with NAFLD has been rarely reported, not to mention the populations with PCOS.

So far, to our knowledge, this is the first study on RTE for assessment of NAFLD in patients with PCOS as compared to healthy controls.

With rapid development of technologies, RTE has been used in more organs than before. The new RTE system has better resolution than the old devices did. Owing to the lower working frequency, the new probe allows for measurements in deeper locations, e.g., in patients with obesity. Automatic displacement of the liver parenchyma is induced by the heartbeat without any pressure from the operator. There is also a sinus curve beneath the RTE image, and this curve shows accuracy of the measurements (16). These technologies reduce interobserver variability remarkably (28).

Semiquantitative parameters of strain histograms of RTE may offer more information about texture elasticity. This approach is reported to be superior to visual scoring for assessment of target strain. In addition, target size has no effect on the strain histogram (29). There is intrinsic relevance among all the 11 semiquantitative strain parameters. Several authors developed different scoring systems based on the parameters calculated with different formulas, and currently there is no consensus (17,18). In this study, we analyzed only the main parameters including MEAN, AREA, and SD, especially MEAN.

Our research confirmed that MEAN decreases in PCOS with NAFLD, while AREA and SD increase as compared to PCOS without NAFLD and healthy controls; these results are in agreement with other studies (12,16,17,29). This finding may be explained theoretically as follows: a softer tissue can be compressed more easily than a stiffer tissue; thus, the displacement of the reflected ultrasound echoes (MEAN) is smaller in the stiffer tissue. AREA is the ratio of low-strain (stiffer) region values. Higher AREA reflects a relatively uneven and harder region of interest. Histologically speaking, major researchers reported that fibrosis associated with hepatic stiffness could be assessed by RTE (30). Furthermore, some researchers believe that steatosis could also affect liver stiffness. Research by Orlacchio and cols. showed that tissue mean elasticity varies consistently with steatosis. Other researchers also found that the steatosis grade is independently associated with a liver elastographic parameter (29,31–33).

In our study, low MEAN was associated with higher WC, BMI, and lipid and transaminase levels as well as with greater endocrine and metabolic disturbances. ROC curve analysis confirmed that MEAN has a good diagnostic value for elevated aminotransferase. These results corroborated the diagnostic value of semiquantitative strain parameters of RTE for NAFLD in addition to conventional ultrasonography.

We found that PCOS patients without NAFLD also had significantly lower MEAN and higher AREA as compared to controls. The levels of MEAN and AREA in PCOS patients without NAFLD were intermediate between those in PCOS patients with NAFLD and healthy controls. According to multiple linear regression analyses, besides WC and MS, PCOS is also a stand-alone independent factor associated with MEAN or AREA values among the subjects without NAFLD. It seems that strain parameters in RTE may distinguish alterations in the liver parenchyma more sensitively: before visual detection by conventional ultrasonic examination. Further research is needed to confirm this notion.

The lack of histological data from livers is a limitation of this study. Nonetheless, the PCOS patients of reproductive age came to our department mostly regarding fertility or menstrual problems, and the controls for a health check-up. Therefore, it was inappropriate for them to undergo an invasive liver biopsy. Nevertheless, comparison of MRI and RTE during assessment of liver changes between patients with PCOS and healthy controls may provide more information.

In conclusion, semiquantitative RTE analysis is useful for distinguishing PCOS patients with or without NAFLD and controls. The proposed method may be able to reveal liver parenchyma alterations in patients with PCOS more sensitively. More studies are needed on semiquantitative RTE analysis for diagnosis of NAFLD and other diffuse liver diseases in various populations including patients with PCOS.
